# New Quality Control Algorithm Based on GNSS Sensing Data for a Bridge Health Monitoring System

**DOI:** 10.3390/s16060774

**Published:** 2016-05-27

**Authors:** Jae Kang Lee, Jae One Lee, Jung Ok Kim

**Affiliations:** 1 LX Spatial Information Research Institute, 163, Anjeon-ro, Iseo-myeon, Wanju_Gun, Jeollabuk-do 55365, Korea; jaekang.lee@lx.or.kr; 2 Department of Civil Engineering, Dong-A University, Busan 49315, Korea; 3 Institute of Construction and Environmental Engineering, Seoul National University, Seoul 08826, Korea; geostar1@snu.ac.kr

**Keywords:** global navigation satellite system, bridge health monitoring system, detection identification adaption, quality control, deflection monitoring analysis

## Abstract

This research introduces an improvement plan for the reliability of Global Navigation Satellite System (GNSS) positioning solutions. It should be considered the most suitable methodology in terms of the adjustment and positioning of GNSS in order to maximize the utilization of GNSS applications. Though various studies have been conducted with regards to Bridge Health Monitoring System (BHMS) based on GNSS, the outliers which depend on the signal reception environment could not be considered until now. Since these outliers may be connected to GNSS data collected from major bridge members, which can reduce the reliability of a whole monitoring system through the delivery of false information, they should be detected and eliminated in the previous adjustment stage. In this investigation, the Detection, Identification, Adaptation (DIA) technique was applied and implemented through an algorithm. Moreover, it can be directly applied to GNSS data collected from long span cable stayed bridges and most of outliers were efficiently detected and eliminated simultaneously. By these effects, the reliability of GNSS should be enormously improved. Improvement on GNSS positioning accuracy is directly linked to the safety of bridges itself, and at the same time, the reliability of monitoring systems in terms of the system operation can also be increased.

## 1. Introduction

Despite significant achievements by numerous researchers in the field, the establishment of Bridge Health Monitoring Systems (BHMS) based on Global Navigation Satellite Systems (GNSS) has been limited due to the reliability issues of GNSS positioning solutions. The information provided by GNSS is based on both the received data and the processing of the data and both are critical for obtaining proper information. If any apparent deflection from GNSS position solutions is detected, it is necessary to verify that it is, in fact, a real deflection behavior resulting from external loading, or if it is an outlier from the data itself. This is an important part from the monitoring system operation perspective. For the former case, potential structural defects can be incurred that pose a great threat to bridge users and to the safety of the bridge itself. For the latter case, false information transfer due to outliers cannot be avoided, thereby having an adverse effect on the reliability of the total monitoring system. In particular, the latter case is a potential risk factor for a GNSS-based BHMS. In line with such a trend, the importance of the GNSS-based BHMS has been emphasized more than ever. Hence, it is essential to consider the accuracy and reliability of GNSS positioning solutions to construct GNSS-based monitoring systems. This investigation aims to address these issues by implementing a quality control algorithm to detect and identify the potential outliers within the data, thereby enabling credible data to be provided to both bridge users and managers.

## 2. Quality Control Algorithm and Quality Measures

The quality of information from a monitoring scheme is typically characterized by its precision, reliability, sensitivity, and economy [1]. In this investigation, monitoring analysis is implemented through a GNSS positioning solution. Accordingly, the precision and reliability of this solution, which can directly affect the monitoring system’s performance, must be discussed. The question to be addressed is whether all GNSS measurements can be converged as a normal distribution by applying least squares estimation to the GNSS measurements. If not, there is a significant possibility that any existing outliers are within a normal distribution range. To assess the precision and reliability of the final GNSS positioning solution, errors should be screened, or other prior-variance calculated, with the remaining measurements; moreover, these errors should be assigned to the adjustment procedure. To estimate the GNSS positioning solution, the two mathematical models mentioned previously are considered. However, outliers cannot be simply considered through functional and stochastic models, because the position accuracy should be assessed with regard to the true value or most probable value (MPV). In processing the GNSS data, systematic errors that were not considered in the functional model and outliers should be isolated before conducting the final least squares estimation to maximize the positioning accuracy and to secure the reliability of the positioning solution. Such a series of inquiries and answers are typically analysed through statistical testing and the answers can thereby be determined. This process is required because of the high sensitivity of the least squares estimation to outliers, which can dangerously reduce the quality of the positioning solution. At this stage, the monitoring analysis quality is vaguely associated with the concepts of precision and accuracy from the GNSS positioning solutions. Therefore, by optimal estimation, an accurate positioning solution can be achieved when proper quality control exists, thereby ensuring that the outliers are excluded.

### 2.1. Least Squares Estimation and Outliers

For detecting and eliminating outliers, a quality control algorithm, specifically, the Detection, Identification and Adaptation (DIA) technique, was deployed in this investigation. Teunissen at Delft University systemized DIA [2,3]. The definitions or concepts of statistics terms used in this study follow the theory of Baarda, who first proposed this algorithm [4]. Prior to the description of the DIA technique, the relationship between the least squares estimation and outliers is briefly examined.

The linearized Gauss–Markov model using dual frequency data can be expressed as follows:

*L* = *Ax* + *v* (1)

The adjusted unknown parameters ($\hat{x}$) are obtained through least squares estimation as follows:

(2) $$\hat{x}={\left(A^{T}PA\right)}^{-1}A^{T}Pl$$

where *A* denotes the design matrix, *P* represents the weight matrix, and *L* is the measurement matrix, and *v* is the residual, and:

(3) $$C_{x}=\sigma^{2}{\left(A^{T}PA\right)}^{-1}={\left(A^{T}C_{l}^{-1}A\right)}^{-1}$$

where $C_{x}$ is the covariance matrix of unknown parameters.

From this, the residuals are estimated as:

(4) $$v=A\hat{x}+l\;with\;P=\sigma_{0}^{2}C_{l}^{-1}$$

(5) $$\hat{v}=\left(I-A{\left(A^{T}PA\right)}^{-1}A^{T}P\right)=J\cdot l$$

(6) $$J=I-A{\left(A^{T}PA\right)}^{-1}A^{T}P$$

The covariance matrix of residuals $C_{v}$ is singular:

(7) $$C_{v}=\sigma_{0}^{2}\left(P^{-1}-A{\left(A^{T}PA\right)}^{-1}A^{T}\right)=\sigma_{0}^{2}\cdot J\cdot P^{-1}$$

From Equation (7), the adjusted parameter can be derived:

(8) $$C_{\hat{l}}=\sigma_{0}^{2}\left(A{\left(A^{T}PA\right)}^{-1}A^{T}\right)$$

From Equations (7) and (8), $C_{v}$ is rearranged:

(9) $$C_{v}=C_{l}-C_{\hat{l}}$$

Equation (6) is then rearranged:

(10) $$J=\frac{1}{\sigma_{0}^{2}}C_{v}P=Q_{v}P$$

where $C_{v}$ is the covariance matrix of covariance.

Thus, the posteriori variance is:

(11) $$\sigma_{0}^{2}=\frac{v^{T}Pv}{n-m}$$

where v is the vector of n residuals, and m is the number of unknown parameters.

At this point, an explanation regarding Equation (9) is warranted. Matrix $C_{l}$ has only diagonal elements, which means that measurements are uncorrelated but residuals are correlated. In other words, a variation of one measurement affects only one unknown parameter and the corresponding residual. At this stage, the relationship between residuals and outliers is required for further consideration. First, true error ε and residual v can be defined as:

(12) $$v=Q_{v}P\varepsilon$$

where it is assumed that each measurement true error $\varepsilon_{i}$ consists of one random error. $\varepsilon_{r_{i}}$ and one outlier, $\nabla l_{i}$:

(13) $$\hat{\varepsilon }=\varepsilon_{r}+\nabla l$$

(14) $$v=Q_{v}P\varepsilon_{r}+Q_{v}P\nabla l=v_{r}+\nabla v$$

where:

$v_{r}=Q_{v}P\varepsilon_{r}$ is only influenced by the random error of residuals, and
$\nabla v=Q_{v}P\nabla l$ is affected by outliers of residuals.

Through this expression, it can be argued that residuals can affect the random error and outlier. The least squares estimation cannot distinguish either of them. Consequently, after conducting the adjustment process, outlier detection is very difficult to perform through the evaluation of the residuals. The stochastic model used for the least squares process is simple; only the variation of estimated coordinates is attained through the model [5]. If the covariance matrix can be obtained from the residuals, it can then be inverted; accordingly, the observational error can be directly calculated. Unfortunately, the J matrix is singular; therefore, the most realistic way to detect the presence of outliers is to test derived quantities, which are functions of them. Details are introduced in the following sub-sections. The main point is that the residuals do not reflect the quality of the measurements; they are not consistently good indicators of outliers. To address this problem, the commonly used DIA technique is applied.

### 2.2. Quality Control Algorithm Procedure

#### 2.2.1. Detection

As mentioned previously, when it is assumed that a systematic error is eliminated from measurements and no outliers exist, the residuals of measurements estimated through least squares estimation converge as a normal distribution, whereas if outliers exist, they are related to the variance estimated from the measurements. The variance, which is estimated from the measurements, including those of the outliers, therefore plays an important role in detecting the outliers. For this process, an F-distribution is used.

The F-distribution consists of an a posteriori variance factor and variance ratio of the population (statistic: ${\hat{\sigma }}_{0}^{2}/\sigma_{0}^{2}$). This is also called the global test on the variance factor (*VF*). It is applicable only when the a priori information on the precision of measurement is available and hypothesis testing is applied for the positioning solution, which has been through a previous adjustment. To this end, hypothesis testing is conducted. The relation between the F-distribution and chi-square is as follows:

(15) $$F_{r,\infty }=\chi_{r}^{2}/r$$

(16) $$\text{Null hypothesis}\;H_{0}:\sigma_{0}^{2}={{\hat{\sigma }}_{0}^{2}}_{v}$$

(17) $$\text{Alternative hypothesis}\;H_{a}:\sigma_{0}^{2}\neq {\hat{\sigma }}_{0}^{2}$$

where $\sigma_{0}^{2}$ is the variance factor and ${\hat{\sigma }}_{0}^{2}$ is the a posteriori variance factor.

When this test is used for the detection of outliers, it is usually expected that ${\hat{\sigma }}_{0}^{2}>\sigma_{0}^{2}$ with regard to most outliers induced by GNSS positioning. Therefore, the null hypothesis test is:

(18) $$H_{0}:\sigma_{0}^{2}={\hat{\sigma }}_{0}^{2}\;under\;H_{a}:\sigma_{0}^{2}>{\hat{\sigma }}_{0}^{2}$$

A one-tailed test is recommended, where:

(19) $$\frac{{\hat{\sigma }}_{0}^{2}}{\sigma_{0}^{2}}<F_{r,\infty ,1-\alpha }$$

Substituting Equation (19) into Equation (20), we can summarize :

(20) $$\frac{{\hat{\sigma }}_{0}^{2}}{\sigma_{0}^{2}}=\frac{v^{T}Pv}{r\sigma_{0}^{2}}$$

where:

(21) $$v^{T}C_{l}^{-1}v<rF_{r,\infty ,1-\alpha }=\chi_{r,1-\alpha }^{2}$$

If the variance factor exceeds the test limit (rejection criteria), the adjustment model is considered invalid. The variance factor test may fail because of input or programming errors, and the presence of outliers in the measurements or model errors, or with poor estimation of the a priori covariance matrix. In this study, it is assumed that the variance factor test fails only under the presence of outliers because the variances of the measurements are known. Assuming that outlier $\nabla l_{i}$ exists, an alternative hypothesis can be written, and the linearized adjustment model may be defined as:

(22) $$A\hat{x}+e_{i}\nabla l_{i}=l+\hat{v}$$

where:

A is the n×m design matrix,

$\hat{x}$ is the vector of m least squares parameter estimates,

l is the vector of n measurements,

$\hat{v}$ is the vector of n least squares residual estimates and

$e_{i}$ is a unit vector in which the $i^{th}$ component has a value equal to 1 and dictates the measurement to be tested:

(23) $$e_{i}=\left[0\;0\;\ldots 1\ldots 0\right]$$

We can express this by the probability distribution of the observation vector l as follows:

(24) $$\text{Null hypothesis}\;H_{0}:E\left[l|H_{0}\right]=Ax$$

(25) $$\text{Alternative hypothesis}\;H_{a}:E\left(l|H_{a}\right)=Ax+e_{i}\nabla l_{i}=E\left(l|H_{0}\right)+e_{i}\nabla l_{i}$$

Under the null hypothesis, the expectation of the residuals is 0:

(26) $$E\left[v|H_{0}\right]=0$$

Therefore, the alternative hypothesis is:

(27) $$E\left(\nabla v|H_{a}\right)=\nabla l_{i}$$

The relationship between ∇l and ∇v from the previous equation is as follows:

(28) $$\nabla v=Q_{v}P\nabla l$$

In the alternative hypothesis, for measurements containing outliers, the expectation of $\frac{{\hat{\sigma }}_{0}^{2}}{\sigma_{0}^{2}}$ can no longer be 1.

(29) $$E\left[\frac{{\hat{\sigma }}_{0}^{2}}{\sigma_{0}^{2}}|H_{a}\right]=E\left[\frac{{\hat{\sigma }}_{0}^{2}}{\sigma_{0}^{2}}|H_{0}\right]+\nabla \left(\frac{{\hat{\sigma }}_{0}^{2}}{\sigma_{0}^{2}}\right)$$

where the variance ratio containing the outlier is redeployed as:

(30) $$\nabla \left(\frac{{\hat{\sigma }}_{0}^{2}}{\sigma_{0}^{2}}\right)=\frac{\nabla {\hat{\sigma }}_{0}^{2}}{\sigma_{0}^{2}}=\frac{1}{\sigma_{0}^{2}}\frac{\nabla v^{T}P\nabla v}{r}=\frac{\lambda }{r}$$

(31) $$E\left[\frac{{\hat{\sigma }}_{0}^{2}}{\sigma_{0}^{2}}|H_{a}\right]=1+\frac{\lambda }{r}$$

where:

(32) $$\lambda =\frac{1}{\sigma_{0}^{2}}\nabla v^{T}P\nabla v=\frac{1}{\sigma_{0}^{2}}\nabla lPQ_{v}P\nabla l$$

and λ denotes a non-centrality parameter.

Under the alternative hypothesis, statistic $\frac{{\hat{\sigma }}_{0}^{2}}{\sigma_{0}^{2}}$ has a non-central $F_{r,\infty ,\lambda }$ distribution with the above non-centrality parameter, λ. This can be converted by the non-centrality boundary value $\left(\lambda_{0}\right)$. Moreover, it can be determined by establishing a different value of confidence level and power of test, depending on the application purpose. According to Cross *et al.* [6], the level of confidence can be different depending on each application, while the power of test is normally set to 80%:

(33) $$\lambda_{0}=\lambda \left(\alpha ,\beta ,r,\infty \right)$$

If the least squares do not converge with the values determined by α and β, it can be said that the measurements may have outliers. $H_{a}$ is dependent on the relationship between residuals and gross ∇l defines simpler and more specific values; therefore, the boundary values should be estimated for vector ∇l when a process is conducted at a given probability level β; $H_{a}$ introduces unidimensional tests on the residuals.

#### 2.2.2. Identification

An outlier, detected through the previously described process, is identified using the w-test data snooping technique [4]. To identify which satellite measurements are responsible, this stage is implemented with new alternative hypothesis testing, which has a standard normal distribution under the null hypothesis. However, under the alternative hypothesis, the distribution of the statistic will have non-centrality. In other words, the w-test for outlier $\nabla l_{i}$ relies on the null hypothesis, which shows that the measurements are outlier-free, as well as on the alternative hypothesis which, when proved true, indicates the existence of an outlier of magnitude ∇l:

(34) $$\text{Null hypothesis}\;H_{0}:E\left[\nabla {\hat{l}}_{i}\right]=0$$

(35) $$\text{Alternative hypothesis}\;H_{a}:E\left[\nabla {\hat{l}}_{i}\right]=e\nabla l_{i}\neq 0$$

To consider a critical value of $\lambda_{0}$ by the determined α and β, $\lambda_{0}$ is rewritten as follows:

(36) $$\lambda_{0}=\frac{1}{\sigma_{0}^{2}}\nabla_{0}l^{T}PQ_{v}P\nabla_{0}l=\frac{\nabla_{0}l^{2}}{\sigma_{0}^{2}}e^{T}PQ_{v}Pe$$

(37) $$\left|\nabla_{0}l\right|=\sigma_{0}\sqrt{\frac{\lambda_{0}}{e^{T}PQ_{v}Pe}}$$

It is then easy to derive a statistic, which tests the alternative hypothesis $H_{a}$:

(38) $$H_{a}:\nabla_{0}l=e_{i}\nabla_{0}l_{i}$$

(39) $$\left|\nabla_{0}l\right|=\sigma_{0}\sqrt{\frac{\lambda_{0}}{P_{i}{\left(Q_{v}P\right)}_{ii}}}$$

On the basis of the previous information, the one-dimensional (one-tailed) test statistic can test the alternative hypothesis, whereby the w-test is defined by the ratio between the size of the outlier and its variance.

Calculation of the test statistic is as follows:

(40) $$w_{i}=\delta_{i}=\frac{v_{i}}{\sigma_{v_{i}}}=\frac{v_{i}}{\sigma_{0}\sqrt{q_{v_{i}}}}$$

(41) $$w_{i}=\frac{e_{i}^{T}Pv}{\sigma_{0}\sqrt{e_{i}PQ_{v}Pe_{i}}}=\frac{{\left(Pv\right)}_{ii}}{\sigma_{0}\sqrt{{\left(PQ_{v}P\right)}_{ii}}}$$

Fixing the significance level α and the power of test β, the non-centrality parameter can then be estimated.

The test statistic for $w_{i}$ is $N_{1-\alpha /2}\left(0,1\right)$ for the situation where:

(42) $$\left(\left|w_{i}\right|=\frac{v_{i}}{\sigma_{v_{i}}}\right)>\left(N_{1-\frac{\alpha }{2}}\left(0,1\right)=\sqrt{F_{1-\alpha_{0}:1,\infty \left(0,1\right)}}\right)$$

(43) $$\sqrt{\lambda_{0}}=\sqrt{F_{1-\alpha ,1,\infty }}+\sqrt{F_{\beta ,1,\infty }}$$

If the *i*th measurement is a supposed outlier, the test is carried out with respect to each measurement; the greatest value that exceeds the critical value is deemed an outlier and is removed from the model. The test statistic is iterated from the first element of the vector; each element has a value of 1. This method can screen for the existence of any potential outliers in individual measurements. The process is repeated until an absolute value or the greatest value is detected. The w-test is then repeated to determine if any further outliers exist. If another outlier is found, it is removed from the model; moreover, the measurement that was first deemed an outlier is reinstated and the model is retested.

**Figure 1:**
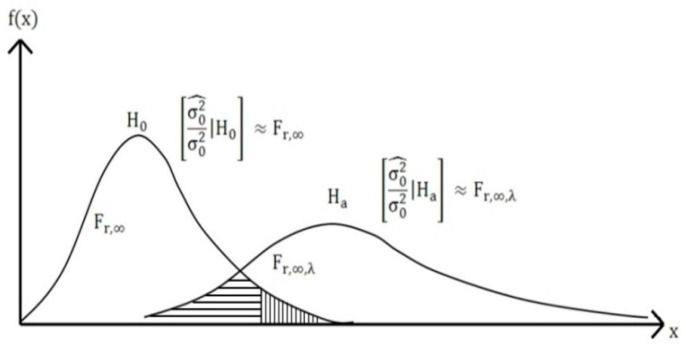
PDF under hypothesis testing.

**Figure 2:**
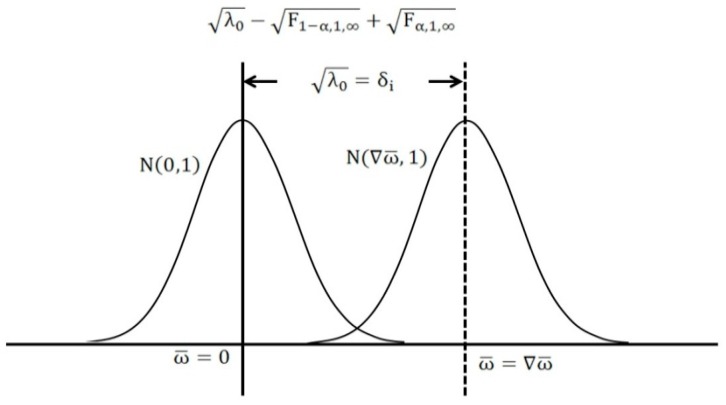
w-test for data snooping.

From Figure 1 and Figure 2, it is evident that, from the significance level and the power of test that the non-centrality parameter can be estimated, $\sqrt{\lambda_{0}}$. This procedure can then be repeated until no further outliers are detected. This type of procedure for screening each individual measurement for the presence of an outlier is known as data snooping.

#### 2.2.3. Adaptation

**Figure 3:**
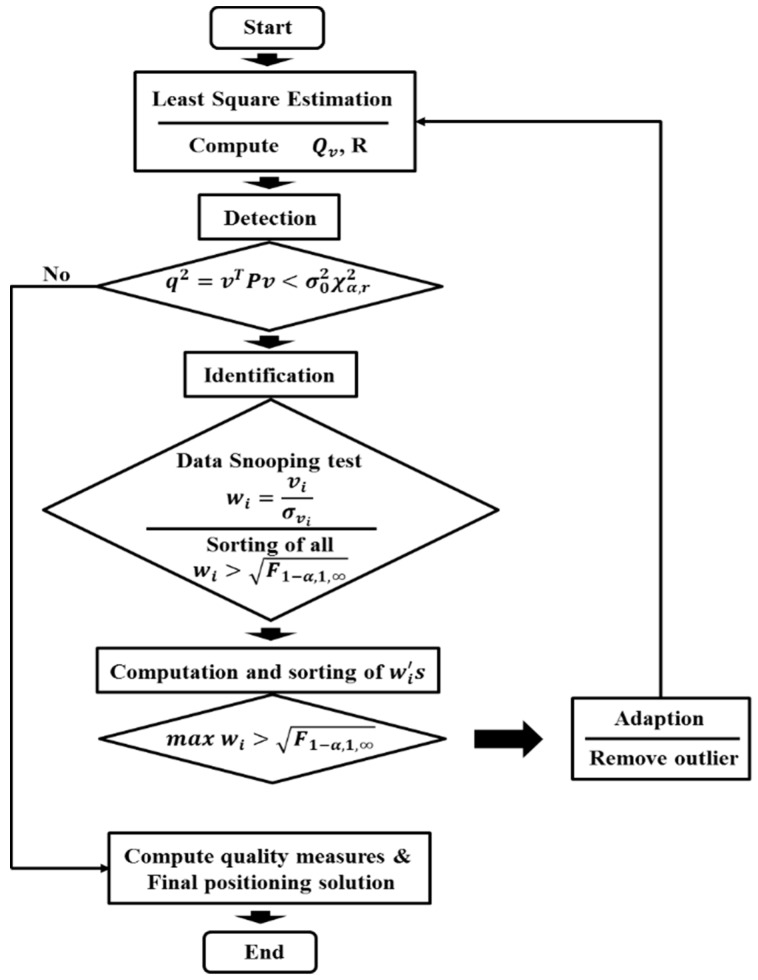
Quality control flowchart.

To facilitate adaptation of the null hypothesis through estimation using the least squares method, the adaptation step is required to remove measurements, including outliers, which are identified through the data snooping technique. Figure 3 depicts the whole of the quality control procedure.

### 2.3. Quality Measures

The quality control outcome is decided on the basis of the number of redundant measurements and the geometry of the satellites. At this stage, the impact of the two factors on the quality control procedure should be checked, as well as the quality of the measurements. The indications of confidence for quality control are redundancy, correlation, and quality dilution of precision (QDOP).

#### 2.3.1. Redundancy Number

Equation (5) illustrated that both random errors and outliers affect the residuals of measurements contained in the outliers. Unfortunately, these two errors cannot be differentiated by the least squares method, which is limited to random error. Therefore, it is difficult to detect outliers by using residuals estimated by the least squares method. However, J = $Q_{v}w$ has a singular and idempotent matrix structure. With this matrix, it is possible to check for the redundancy number if the sum of the diagonal elements of the matrix (known as the trace of the matrix) is the same as the degrees of freedom:

(44) $$Trace(Q_{v}w)=rank(Q_{v}P)=n-u=r$$

where r is the total redundancy. If $r_{i}$ denotes the diagonal elements of J = $Q_{v}P$, from Equation (6), $r_{i}$ is redefined as follows:

(45) $$\overset{n}{\underset{i=1}{\sum }}r_{i}=rr_{i}={\left(Q_{v}P\right)}_{ii}=Q_{v_{i}}P_{i}=e_{i}^{T}Q_{v_{i}}P_{i}e_{i}$$

Because of the nature of the idempotent matrix, the elements of this matrix are distributed in the range of $0\leq r_{i}\leq 1$. The closer the redundancy is to 1, the closer the variance of residuals is to the variance of the `measurements. Thus, in conclusion, this relation is the same with random errors of residual and measurements; therefore, the estimated coordinates (GNSS positioning solution) with a high level of precision can be obtained. However, if the redundancy is closer to 0, the variance of residuals has a smaller value close to 0; therefore, this value cannot be a correct comparison with the value of the measurements. In other words, a low level of redundancy is insufficient for separating outliers. Furthermore, the measurements have a higher possibility of containing outliers; moreover, a high level of redundancy means that the outliers can be easily detected because the outliers have an impact on the residual. Nevertheless, if the redundancy becomes 0, the outliers cannot be detected. Therefore, estimated coordinates may not be accurate. In general, when the level of redundancy is higher than 0.5, it can be considered well measured [6]. If ∇v is reformed through Equation (46), it can be expressed as $\nabla \bar{v}=Q_{v}w\nabla l$, and the individual $\nabla v_{i}$ can be defined as:

(46) $$\nabla v_{i}=r_{i}\cdot \nabla l_{i}$$

This can be seen as the contribution of a single measurement $l_{i}$ against the whole redundancy *r*. Relative redundancy can then be defined as the average of the diagonal elements of *M*:

(47) $$\nabla v_{i}=r_{i}\cdot \nabla l_{i}$$

(48) $$M=Q_{v}w=I-Q_{\bar{l}}w=I-U$$

in which:

(49) $$U=Q_{\bar{l}}w=AQ_{\bar{x}}A^{T}w$$

The U matrix is also an idempotent matrix, which can be described as:

(50) $$Trace\left(U\right)=Rank\left(U\right)=Rank\left(Q_{x}\right)=U$$

(51) $$U_{i}\cdot \nabla l_{i}=\nabla l_{i}-r_{i}\nabla l_{i},$$

If an outlier ($\nabla l_{i}$) occurs in only one measurement ($l_{i}$), it will be reflected in the corresponding residual ($v_{i}$), as much as $\nabla v_{i}=r_{i}\nabla l_{i}$. Hence, it is now clear that an outlier ($\nabla l_{i}$), coupled with a large number $r_{i}$, will more greatly affect the corresponding $v_{i}$.

#### 2.3.2. Quality Dilution of Precision (QDOP)

QDOP is a quality measure that determines how much influence the geometric constellation of satellites has on internal reliability and redundancy. By using the redundancy represented in Equation (50), this can be rearranged as:

(52) $$r_{i}=\left(P^{-1}-AQ_{\hat{x}}A^{T}\right)P=1-A{\left(A^{T}PA\right)}^{-1}A^{T}P$$

In this equation, a coefficient matrix representing the geometry of the tracking satellites enables the precision of measurement to be expressed as the QDOP value:

(53) $$QDOP_{i}=e_{i}^{T}A{\left(A^{T}A\right)}^{-1}A^{T}e_{i}$$

QDOP is in the range of 0 to 1. It is evident that if the measurement estimated from a satellite constellation with good geometry is closer to 0, the ability to detect an outlier can be enhanced because the redundancy was increased and the minimal detection bias (MDB) was decreased. However, if QDOP is closer to 1, in the relation between internal reliability and redundancy, the redundancy is closer to 0. This is because the denominator of the internal reliability is also 0 and the result of the MDB is infinity. The coefficient matrix then becomes the non-singular matrix. In other words, to achieve a higher redundancy, QDOP should be minimized [7].

#### 2.3.3. Reliability

Deformation analysis involves two types of errors: measurement and deformation models [1]. To avoid misinterpretation of systematic errors or outliers in the measurements as deformation phenomena, screening of the measurements for outliers or systematic errors should be performed prior to the estimation of the deformation parameters. The concept of reliability originates from Baarda [4]. Generally, reliability assesses the capability of detecting outliers. In this respect, “internal reliability and external reliability” are distinguished. The internal reliability of a GNSS positioning solution is its ability to detect outliers by testing a hypothesis made with a specific confidence level (1 − α) and the power of test (1 − β). In other words, higher reliability means that even small errors can be found, whereas lower reliability means that even larger errors cannot be detected. The other way of testing is through the external reliability of the GNSS positioning solution in informing of the impacts of undetected errors against a detected unknown vector or estimated coordinates. Hence, high external reliability means that the undetected errors from the quality control procedures have minimal impact on coordinates. The number of redundancy measures, the geometry of the tracking satellites, and error propagation all affect reliability. Therefore, if numerous redundant measurements or many tracking satellites exist, the reliability should improve.

- *Internal reliability* 

Internal reliability refers to the maximum number of undetectable errors and is simply represented by MDB. This variable is assessed by the lower values $\nabla_{0}l_{i}$ of outlier $\nabla l_{i}$, which can detect, through the data-snooping test, if any remaining errors heavily affect the final estimation of coordinates. MDB is the magnitude of the smallest bias detectable and is determined for a given confidence level. The critical value mentioned earlier can be simplified by diagonal elements of the weight matrix P (Q):

(54) $$\left|\nabla_{0}l_{i}\right|=\sigma_{0}\frac{\lambda_{0}}{\sqrt{P_{i}{\left(Q_{v}P\right)}_{ii}}}=\sigma_{0}\frac{\lambda_{0}}{\sqrt{P_{i}r_{i}}}$$

According to this formula, the detectable outlier directly depends on the precision of the measurement or its variance ($\sigma_{l_{i}}$). Outliers occur when it is in the significance level of α in type 1 errors and β in type 2 errors.

(55) $$\left|\frac{v_{i}}{\sigma_{v_{i}}}\right|\geq N_{\alpha +\beta }$$

The power of test was suggested by Cross [6] to be typically 20%. The result of the previous two rearranged equations is the following:

(56) $$\left|MDB\right|\geq \frac{\lambda_{0}\sqrt{Q_{v}}}{Q_{v}P}=\frac{\lambda_{0}\sqrt{Q_{v}}}{r}$$

According to this equation, MDB is simultaneously dependent on the redundancy number and the covariance of the residuals, not on the measurement and residual.

- *External reliability* 

External reliability relates to the maximum effect of a possibly undiscovered observational outlier on the estimates of unknown parameters. It verifies whether an undetected error exists, and what impact the error would have on estimated coordinates [6]. Thus, external reliability can show the impact of MDB on estimated coordinates:

(57) $$\nabla_{0}\hat{x}={\left(A^{T}PA\right)}^{-1}A^{T}e_{i}P\cdot \left(MDB\right)=Q_{\hat{x}}A^{T}Pe_{i}\cdot \left(MDB\right)$$

where $Q_{\hat{x}}$ is the posterior variance-covariance matrix of the estimated parameters.

## 3. Bridge Experiment

### 3.1. Introduction

In the application of a BHMS-based GNSS, monitoring is proactively performed and is completely dependent on the GNSS positioning solution. The developed quality control algorithm should therefore be applied to improve the reliability of the monitoring system. To test it, the authors applied the developed algorithm to GNSS data collected from an in-service bridge, the SeoHae cable-stayed bridge located in South Korea, which is a test-bed on which the application of GNSS has already been demonstrated [8,9]. However, although previous investigations have involved improvement of the GNSS solution, case studies on removing outliers to improve the accuracy of a GNSS solution have rarely been performed.

#### 3.1.1. Evidence of Existing Outliers

A long-span bridge that is built for expanding trade, transport, and logistics, and which can be used by a number of different vehicles, often interferes with GNSS signals because of the usual amount of passing vehicles, the bridge structure itself, and environmental factors.

**Figure 4:**
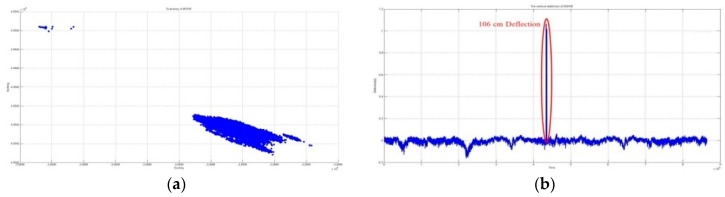
Three-dimensional coordinates of the point of MSWE. (**a**) Horizontal coordinate at MSWE including outliers; (**b**) Vertical coordinate at MSWE including outliers.

**Figure 5:**
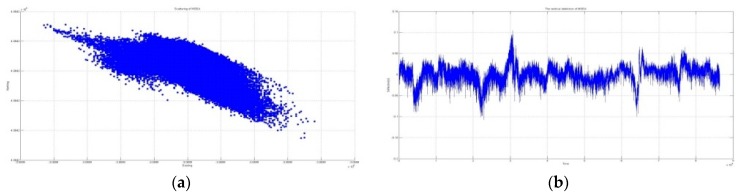
Three-dimensional coordinates of the point of MSEA. (**a**) Horizontal coordinate at MSEA excluded outlier; (**b**) Vertical coordinate at MSEA excluded outlier.

Figure 4 and Figure 5 graphically depict the results of vertical deflection, estimated from two GNSS receivers installed at both sides of the mid-span such that, in the lateral direction, the two receivers were installed at the same position. It is normal that no significant difference occurred in the amount of vertical deflection and its trend, as shown in the figures.

#### 3.1.2. Test Description & Test-Bed

- *Test Description* 

**Figure 6:**
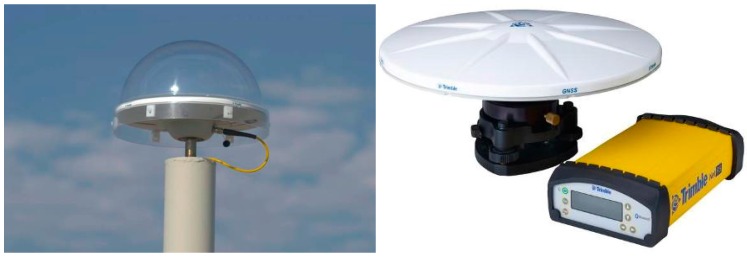
Antenna of the reference station and rover.

To conduct this research, GNSS data was collected through an array of six receivers for 24 h from 09:00 on 25 March 2010. Data was received at a sampling rate of 1 Hz (1 s). The antenna used at the reference station and rovers was a Zephyr Geodetic Model 2 produced by Trimble Navigation, Ltd., (Sunnyvale, CA, USA, Figure 6 left). Receivers installed on the bridge as a rover were identical, *i.e.*, the same model, namely a Trimble NetR5, produced by Trimble Navigation, Ltd., (Sunnyvale, CA, USA, Figure 6, right). These data were initially received at the GNSS receiver before being transmitted to the SeoHae Bridge management office through a wireless modem. The positioning methodology employed for processing GPS data from bridge is single baseline data processing which is commonly used for BHMS based GNSS. Details are provided in Roberts *et al.* [10].

- *Test-Bed* 

**Figure 7:**
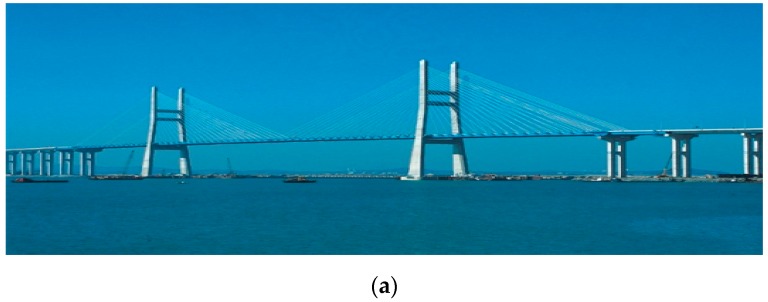

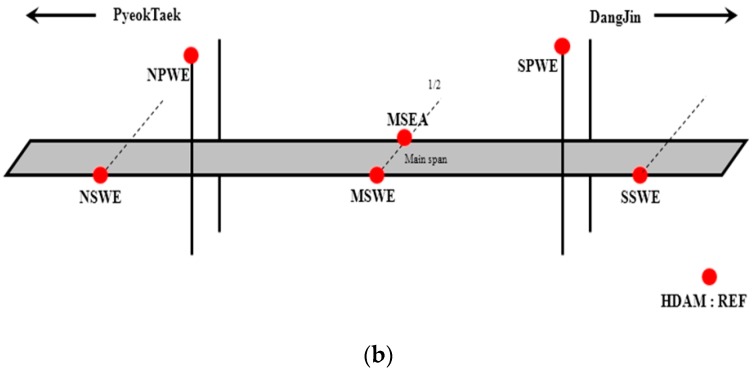
SeoHae cable-stayed bridge [8]. (**a**) Photographs of the SeoHae cable-stayed bridge; (**b**) Location of the GNSS receiver and name of station.

The SeoHae cable-stayed bridge Figure 7), which is selected by the test-bed of this research, is part of the total length of the 7.31-km SeoHae Bridge. It is a continuous bridge that is composed of a steel box girder with five spans totaling 990 m (60 + 200 + 470 + 200 + 60). From the point of the main span, it is the second longest cable-stayed bridge in service in Korea following the Incheon cable-stayed bridge.

#### 3.1.3. Pre-Analysis on the Status of GPS Signal

**Figure 8:**
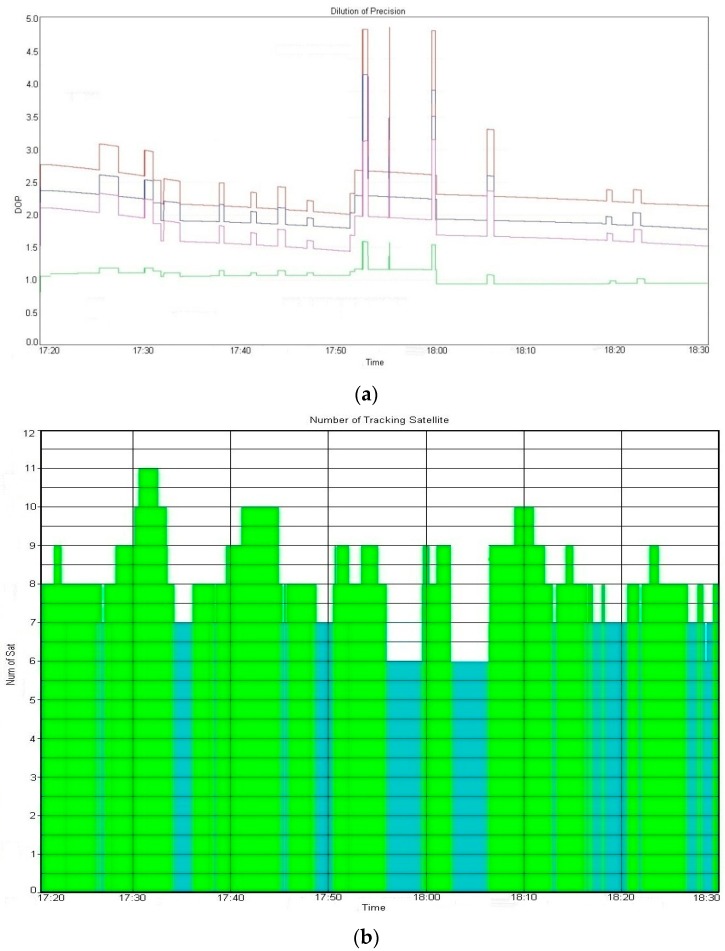
DOP values and number of satellites. (**a**) DOP value; (**b**) The number of tracking satellites.

To apply the developed quality control algorithm, we used a 5% significance level and 80% power of test. During the observation session, some previously defined outliers occurred for approximately 7 min (05:52 to 05:59 p.m.). To detect and eliminate the outliers, we extracted approximately 2 h of data based on the time when the outliers occurred. Figure 8 helps describe some of the DOP values and the number of tracking satellites used during the observation session. 

It is well known that the number of tracking satellites is integrally related to the DOP value, however, any change in the number of tracking satellites can likewise affect the subsequent analysis of a variety of quality measures.

### 3.2. Quality Measures

GNSS data obtained from the in-service bridge contained a number of errors that were considered outliers. Through the application of the developed quality control algorithm, a number of DIA procedures were applied that affected the outliers; these procedures might have minimized coordinate errors in the final GNSS positioning solution. In this process, the validation of the algorithm and the effect of outlier removal can be explained by the quality measures described previously. This enables confirmation that we obtained an enhanced GNSS positioning solution.

#### 3.2.1. QDOP and Redundancy

**Figure 9:**
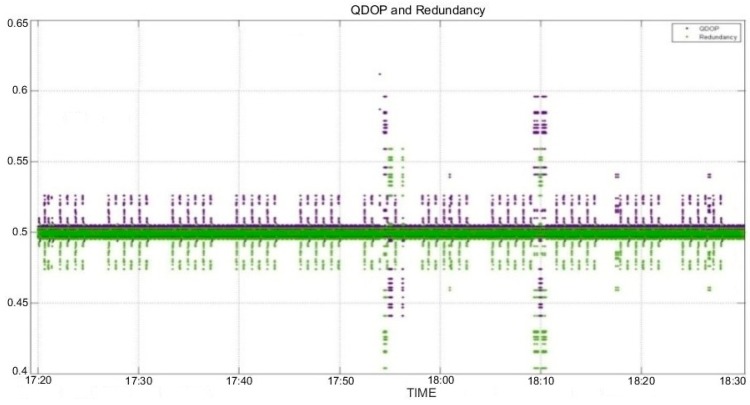
QDOP and redundancy.

The first considered quality measures were QDOP and redundancy, as shown in Figure 9. The number of changes made to the tracking satellites affected QDOP and redundancy. In the section with a reduced number of satellites, QDOP values were increased and redundancy was decreased. The relationship between the change in the number of tracking satellites and redundancy likewise showed a similar trend; it was determined that QDOP had the opposite trend.

#### 3.2.2. Analysis on Posterior Variance

**Figure 10:**
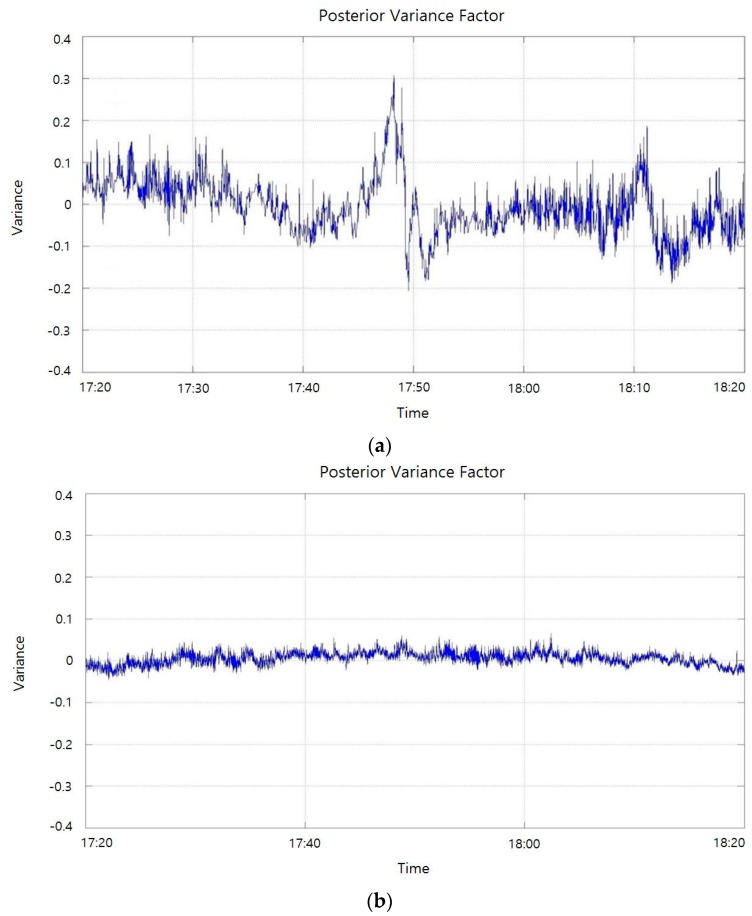
Posterior variance with QC and without QC. (**a**) Posterior variance without QC; (**b**) Posterior variance with QC.

As shown in Figure 10, posterior variance was significantly stabilized or decreased by applying the developed quality control algorithm.

#### 3.2.3. Reliability

**Figure 11:**
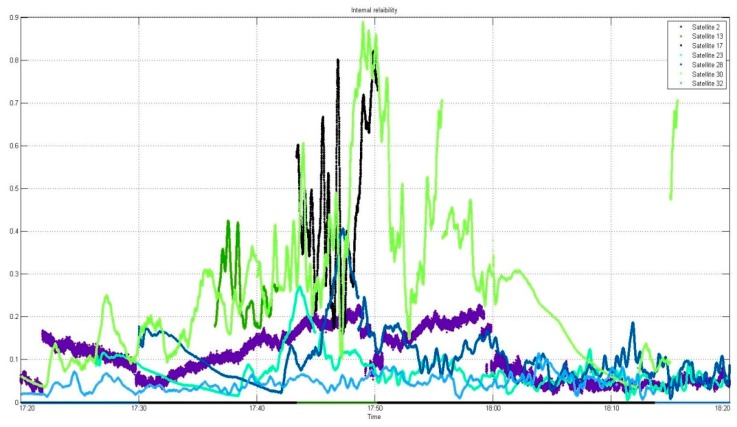
Analysis of internal reliability.

**Figure 12:**
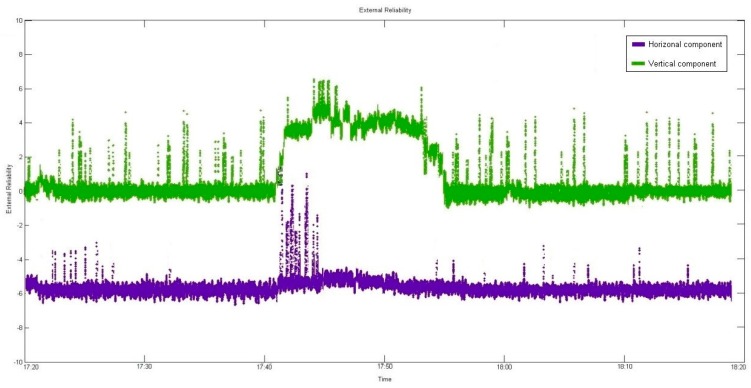
Analysis of external reliability.

After applying the developed algorithm for quality control, the reliability analysis was divided into external and internal reliability analyses, as shown in Figure 11 and Figure 12, which show the respective results of internal and external reliability. Despite elimination of the outlier, it is evident that its influence remained.

- *Internal reliability* 

- *External reliability* 

### 3.3. Improved Accuracy of GNSS Positioning Solution

The aim of this sub-section is to demonstrate that the application of the developed algorithm for quality control increased the accuracy of the horizontal and vertical positioning.

#### 3.3.1. Enhanced Vertical Positioning

**Figure 13:**
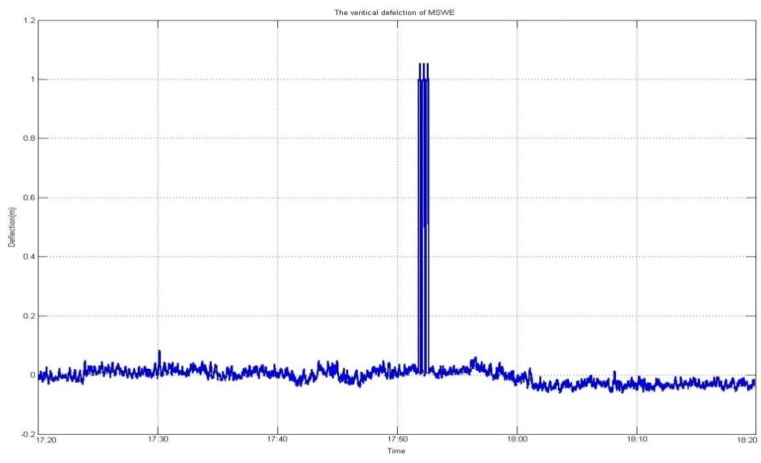
QC not applied to height coordinate.

**Figure 14:**
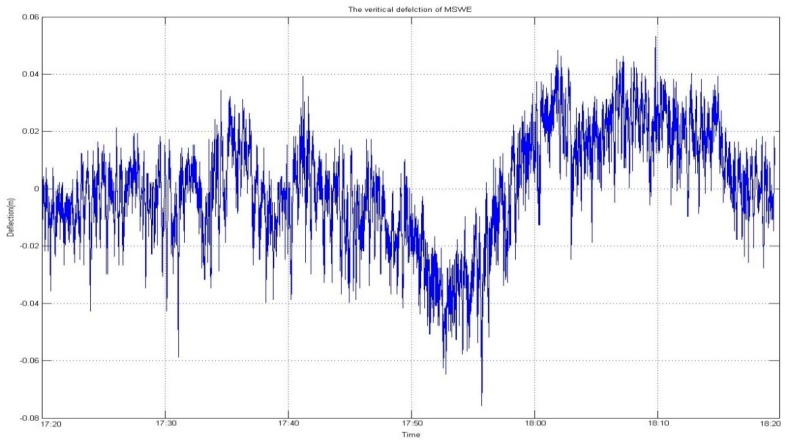
QC applied to height coordinate.

With regard to height, a GNSS receiver installed on a bridge can assess the deflection of the deck. This deflection analysis provides information on the changed time-series of vertical deflection to the BHMS. However, if the positioning solution is contained in outliers, the delivery of false information cannot be avoided. Therefore, in implementation of the BHMS, accurate and reliable delivery of information on vertical deflection is critical. Figure 13 and Figure 14 show the results of the comparison before and after applying the developed algorithm for quality control. As shown in the figures, a more normal deflection was monitored with a more significantly reduced RMS due to the elimination of the outliers. Table 1 summarizes the results.

#### 3.3.2. Horizontal Direction Positioning Solution

**Figure 15:**
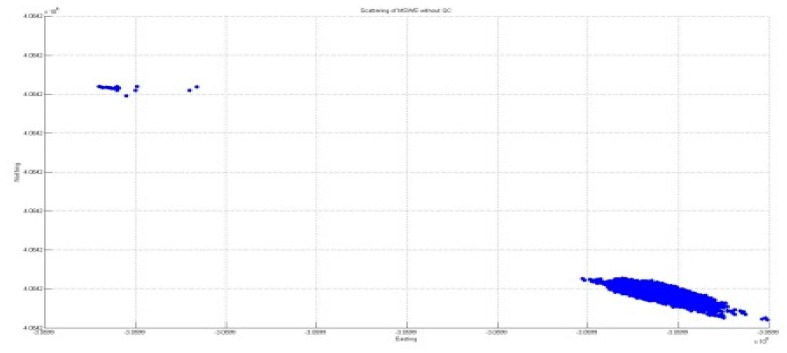
Horizontal positioning solutions without QC.

**Figure 16:**
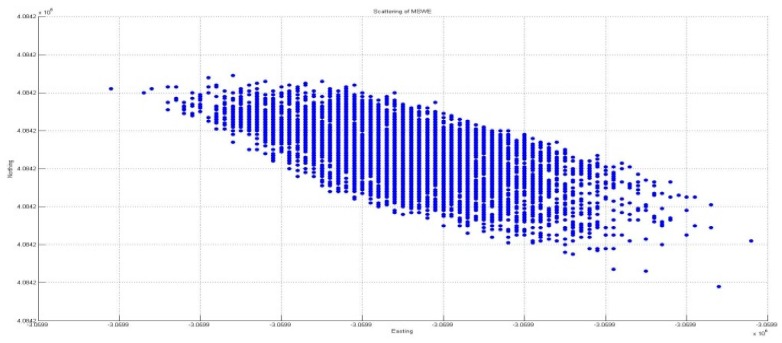
Horizontal positioning solutions with QC.

Following the increase in accuracy for vertical deflection, the application of the developed algorithm for quality control was considered in terms of horizontal positioning. This position is regarded as the lateral and longitudinal directions, which are also important in the construction of a BHMS because deflection in the lateral direction is normally induced by wind loading; moreover, deflection in the longitudinal direction is used along with pier inclination monitoring. Figure 15 and Figure 16 show the comparison results of the horizontal solutions before and after applying the developed algorithm for quality control.

The figures in Table 2 show that the elimination of outliers was achieved through the developed algorithm for quality control. However, despite applying quality control, seven outliers remained, which accounted for 6.3% of the total 110 outliers. However, this percentage was because the significance level was set to be 5% in the statistical process.

- *Error Ellipse Comparison* 

In general, analysis on the error ellipse is additionally used as a statistical indicator. In this case, the major axis of the error ellipse indicates the direction of the larger errors (*i.e.*, the largest standard deviation); the minor axis indicates the direction of lower errors. The accuracy of the horizontal position in the error ellipse is then represented by considering the correlation between the north-south and east-west directions.

**Figure 17:**
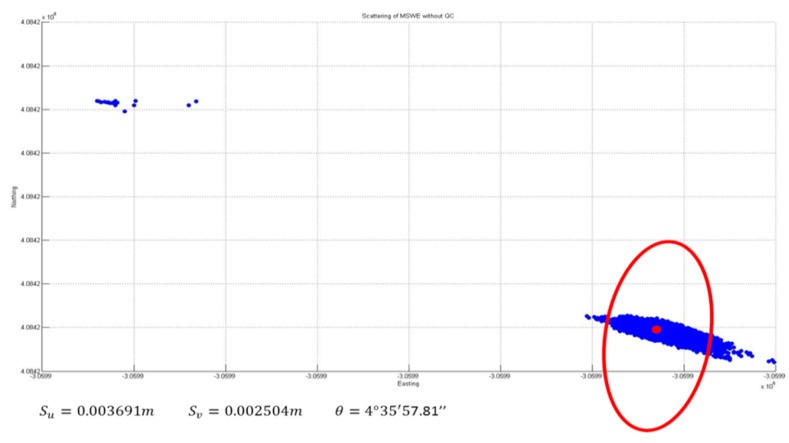
Error ellipse without QC.

**Figure 18:**
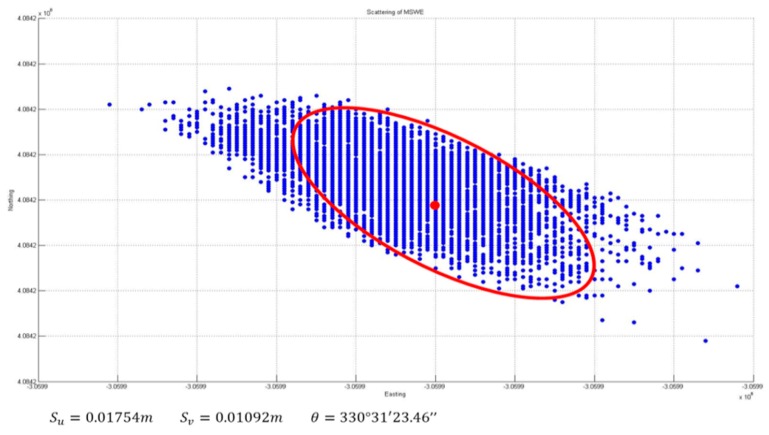
Error ellipse with QC.

Figure 17 and Figure 18 present a comparison of the error ellipses before and after applying the developed algorithm for quality control. Consequently, it was confirmed that the length of the major axis of the error ellipse was reduced by approximately 47.52 mm through the application of quality control. In addition, the azimuth of the error ellipse was changed by 34°; accordingly, it is considered that the deviation of direction occurred because of the outliers.

## 4. Concluding Remarks

This investigation presented an attempt to prove the accuracy of the GNSS positioning solution for use in a BHMS based on GNSS. The applicability of GNSS for BHMS has already been validated through prior research, however, specific problems were exposed in the delivery of a reliable GNSS solution. These problems were due to the lack of consideration of outliers; the occurrence of such outliers can provide false information to the BHMS when the positioning solution is used in deflection and dynamic characteristic analyses, such as for natural frequencies and damping ratios. An experiment was performed on a long-span cable-stayed bridge. The results confirmed that approximately 110 outliers were present in a sample of real data, which had a clear influence on the GNSS positioning solution. However, when the proposed algorithm for quality control was used as a DIA procedure to minimize the effect of outliers, 93.7% (103) of all outliers were eliminated, and the results were shown to be much more reliable.

## Figures and Tables

**Table 1:** Vertical Deflection (unit: m).

	Without QC	Applied QC	Improvement
**Max**	1.055	0.051	483%
**Min**	−0.062	−0.076	122%
**RMS**	0.088	0.018	489%

**Table 2:** Horizontal positioning solution.

		Without QC	With QC
**Easting**	**Max**	−3,059,909.6	−3,059,886.35
	**Min**	−3,059,909.97	−3,059,886.43
**Northing**	**Max**	4,084,206.02	4,084,201.51
	**Min**	4,084,205.42	4,084,201.4
	**RMS**	0.028	0.01 (280%)

**Table 3:** Ellipse error comparison.

	Length of Major Axis	Azimuth
**Without QC**	0.0369 m	4°35′57.81″
**Applied QC**	0.0375 m	330°31′23.46″
**Difference**	47.52 mm	34°4′13.95″
